# Quality of Life Theory I. The IQOL Theory: An Integrative Theory of the Global Quality of Life Concept

**DOI:** 10.1100/tsw.2003.82

**Published:** 2003-10-13

**Authors:** Soren Ventegodt, Joav Merrick, Niels Jorgen Andersen

**Affiliations:** The Quality of Life Research Center, Copenhagen K, Denmark

**Keywords:** Quality of Life, integrative quality of life, IQOL, QOL, SEQOL, QOL5, QOL1, human development, holistic medicine, public health, Denmark, etiology

## Abstract

Quality of life (QOL) means a good life and we believe that a good life is the same as living a life with a high quality. This paper presents the theoretical and philosophical framework of the Danish Quality of Life Survey, and of the SEQOL, QOL5, and QOL1 questionnaires.The notion of a good life can be observed from subjective to the objective, where this spectrum incorporates a number of existing quality of life theories. We call this spectrum the integrative quality-of-life (IQOL) theory and discuss the following aspects in this paper: well being, satisfaction with life, happiness, meaning in life, the biological information system (“balance”), realizing life potential, fulfillment of needs, and objective factors.The philosophy of life outlined in this paper tries to measure the global quality of life with questions derived from the integrative theory of the quality of life. The IQOL theory is an overall theory or meta-theory encompassing eight more factual theories in a subjective-existential-objective spectrum. Other philosophies of life can stress other aspects of life, but by this notion of introducing such an existential depth into the health and social sciences, we believe to have taken a necessary step towards a new humility and respect for the richness and complexity of life.

## INTRODUCTION

This paper presents the theoretical and philosophical framework of the Danish Quality of Life Survey[1,2] and the SEQOL[3], QOL5, and QOL1[4] questionnaires, corresponding to the methodological criterion outlined in another paper[5].

Quality of life (QOL) means a good life. A good life is the same as living a life with a high quality. This may seem evident, but it is necessary to make such a simple clarification, because medical jargon often uses very narrow concepts of the quality of life (for example, side effect profiles). Medical advertisements often depict the quality of life as one factor among many, on a par with other improvements that a certain medical product promises. In this work, the quality of life belongs on the highest, most common level of life.

All great religions and philosophies have a notion of a good life ranging from saying that a good life is attained by practical codes of conduct to requests to engage in a certain positive attitude to life or to search into the depths of your own being. Notions about a good life are closely linked to the culture of which you are a part. When people in a Western culture view a good life, the cultural conditioning makes them tend to include happiness, fulfillment of needs, functioning in a social context, etc. These notions can then be divided into three loosely separate groups, each concerned with an aspect of a good life:

1. The *subjective* quality of life is how good a life each individual feels he or she has. Each individual personally evaluates how he or she views things and his or her feelings and notions. Whether an individual is content with life and happy are aspects that reflect the subjective quality of life.
2. The *existential* quality of life means how good one’s life is at a deeper level. It is assumed that the individual has a deeper nature that deserves to be respected and that the individual can live in harmony with. We might think that a number of needs in our biological nature have to be fulfilled, that these factors — such as conditions of growth — must be optimized, or that we must all live life in accordance with certain spiritual and religious ideals laid down by the nature of our being.
3. The *objective* quality of life means how one’s life is perceived by the outside world. This view is influenced by the culture in which people live. The objective quality of life reveals itself in a person’s ability to adapt to the values of a culture and tells us little about that person’s life. Examples may be social status or the status symbols one should have to be a good member of that culture. (Objective is used here in the sense of nonsubjective or objective facts. Nonsubjective is concerned with the external and easily established conditions of life that many observers can rate identically.)

As these three overall aspects of the quality of life are loosely grouped with notions relevant to the quality of life, which tend to overlap, they can be placed in a spectrum ranging from the subjective to the objective. We place the existential element in the middle, because it unites the subjective and the objective, as shown later. This existential center also represents the depth of the being of humanity (see Fig. 1). Other researchers of the quality of life integrate the subjective and the objective aspects of a person's life at a higher state in life. This has been expressed variously by the terms flow[6] and sense of coherence[7].

This spectrum, from the subjective to the objective quality of life via the quality of life in the existential depths, incorporates a number of existing quality-of-life theories. We therefore call this spectrum the integrative quality-of-life (IQOL) theory. The theories and their main aspects, depicted in Fig. 1, are dealt with in the following paragraphs. Each theory is presented, followed by a short synopsis of the central notion and how it relates to other life theories and notions.

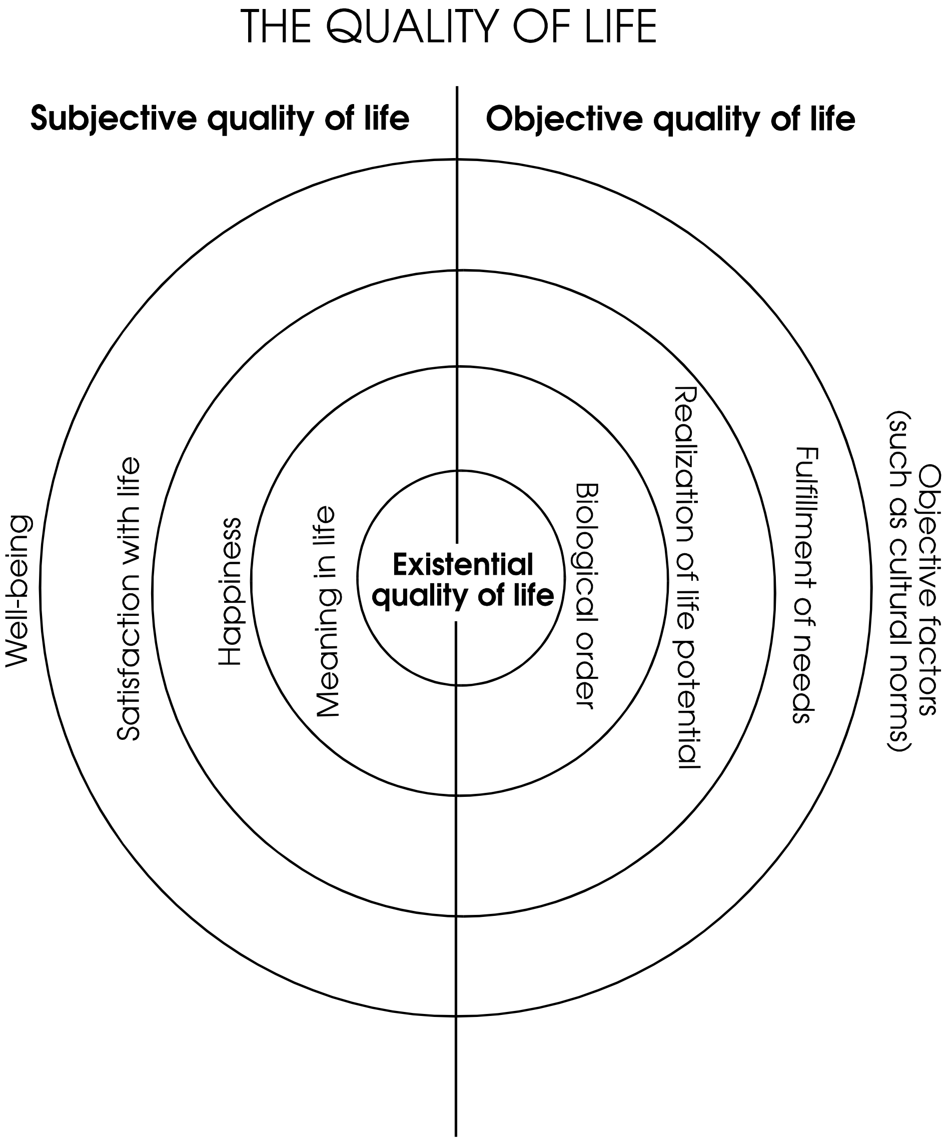

**FIGURE 1.** The integrative theory of the quality of life. The individual can best be compared to a green apple with red patches (a subjective and an objective quality of life, respectively, at the surface of an individual's existence) with a hidden nucleus (humanity's inner depth). When this picture is combined with the picture of humanity as an onion with a number of layers between the surface and the nucleus, the taxonomy underlying the quality-of-life analysis is achieved. Between life's surface and its inexpressible depth lie well being, satisfaction, harmony, and meaning and deep concord.

## THE INTEGRATIVE THEORY OF THE QUALITY OF LIFE

### Well Being

The most natural aspect of the subjective quality of life is well being. The quality of life is seen here in terms of an assessment of one's own quality of life[8]. When we meet other people, we always say, "How are you? or "How's life?" We are thus asking that person to give us an evaluation of their quality of life.

Such questions do not require a lengthy explanation of matters of life, merely a spontaneous assessment of life in general. If, however, we are asked how content we are with life or how happy we are, these questions are assessed differently to such a question as "How are things?"

Such questions are much more complex. In other words, satisfaction with life and happiness are deeper dimensions that are not as straightforward as well being.

The question about well being is followed by an explanation: if we are told that things are not going well, what was just said may typically be extended, as follows: "Things are not going too well at work (home);" "My health is not what it used to be." This means that well being is closely linked to how things function in an objective world and with the external factors of life.

When we speak about feeling good, we do not generally embark on a lengthy discussion of the meaning of life and the deep, existential issues and aspirations we all harbor. Well being is thus something else and more superficial than meaning in life, fulfillment of needs, and self-realization.

Most people tell a lot of people that they feel good, yet only to a very few do people dare open up and take stock of the meaning of our lives. We have a surface we open up to everybody and a hidden depth to which very few people have access, often not even our conscious selves. The spontaneous self-experienced quality of life might seem such a natural thing that it may be the reason why it has not given cause to serious investigation, let alone reflection.

## Satisfaction with Life

When people are asked whether they are satisfied with life, they often say that something or other is amiss. People are usually less satisfied with life than their state of well being would indicate. People tend to feel good, but are not very satisfied, just satisfied. In retrospect, there is always something to be dissatisfied or disgruntled about.

Being satisfied means feeling that life is the way it should be. When one's expectations, needs, and desires in life are being met by the surrounding world, one is satisfied. Satisfaction is a mental state: a cognitive entity.

This symmetry and concord can come about in two ways: either we try to change the external world so that it matches our dreams or we give up our dreams because they are unrealistic, and adapt them to the world as it is, thus creating concord between the external world and our dreams. Both approaches generate the same satisfaction. However, these two strategies of life generate entirely different lives: one life meets with one's dreams and the other life is lived in resignation; but both lives will be satisfactory. Thus, satisfaction does not necessarily involve realizing life potential, fulfillment of needs, or the ability to function well in life objectively. A person who has lived a difficult life, such as prostitution, chronic illness, or poverty, always seems to be satisfied with his or her life because of gradual adaptation through resignation.

One can be satisfied with life yet feel bad inside. The process of adapting to one's environment may lead to an individual letting him- or herself down by compromising the deep dreams of a good life. The person may not be unhappy with life but deep down considers it meaningless. Satisfaction with life is thus not the same as experiencing meaning in life. It is very common to be satisfied yet not happy.

There are classical types of satisfaction theories. One of these is called preference theory. It is typically formulated in such a way that a good life lies in seeing one's wishes come true[9]. "Seeing" because it is not enough that one's wishes be fulfilled. One has to experience that they actually are. This theory leaves the individual free to make his or her choice. One may, for instance, choose to collect stamps or good friends. The quality of life is based on whether one gets what one wants. In relation to this, the World Health Organization (WHO) has defined health broadly as a state of complete physical, mental, and social well being. By stressing well being, that is, the experience of feeling good, the WHO is in accordance with the other theories in these groups.

The theory does not distinguish between constructive and (for example, self-) destructive needs. For example, the wish to die or to hurt or harm others increases the quality of life when it is realized.

Preference theories are a subgroup to the gap theories, which are found in a multitude of forms. Some include time, others the realization of life potential here and now in time and space, etc. They aim to find harmony in what you want life to be and how you think it is. The smaller the gap between them, the greater one's satisfaction, and hence, according to certain theories, the greater the quality of life.

The majority of quality-of-life theories focus on satisfaction. The concept is fairly easy to deal with and, as a cognitive concept, lends itself well to intellectual thought and rumination. Researchers and philosophers prefer it to more emotionally and intuitively attained concepts.

The problem of using (life) satisfaction as the only measure of the quality of life is that a good life is more than merely being satisfied: happiness, meaning in life, fulfilling one's needs, etc.

## Happiness

Most people use this word with caution, because it has special significance. They use it with respect. Being happy is not just being cheerful and content. It is a special feeling that is precious and very desirable, but hard to attain. Happiness is something deep in the individual that involves a special balance or symmetry. Happiness is an intoxication, a rare sweetness of life, when tiny bubbles sparkle. It is best described in metaphors, preferably by poets.

Happiness is closely associated with the body, but is not limited to it. It comprises an individual's whole existence and is signified by a certain intensity of an experience, which is also the case with unhappiness. The intensity of the experience is a dimension that does not separate happiness from more superficial aspects of the quality of life such as being satisfied with life and well being.

Many people link the concept of happiness with human nature: happiness comes to people who live in extraordinary harmony with his or her nature. Nevertheless, not many people believe that happiness is achieved by merely adapting to one's culture and related factors[1]. In other words, happiness requires individuals not to resign too much but fight for what, deep down, is important to them.

Typically, happiness is associated with nonrational dimensions, such as love, close ties with nature, etc., but not with money, state of health, and other objective factors. Happiness is found in classical philosophy and religious concepts, and it has inspired humanity broadly[10].

## Meaning in Life

Meaning in life is a very important concept and is seldom used. We only speak of the meaning of life with our most intimate friends and relatives, if at all. People who seek meaning in life are often catapulted into a confusing situation, where the value of all aspects of life is viewed quite differently. Are relations with my friends or partner as meaningful as they ought to be? Am I doing the right thing in life? Have I got the right job? Am I using my talents in the right way? Are my beliefs in life really correct?

A search for meaning in life involves an acceptance of the meaninglessness and meaningfulness of life and an obligation towards oneself to make amends for what is meaningless. In this way, the question of meaning in life becomes deeply personal, and very few people attempt to answer it because, by doing so, we risk our security in everyday life.

The problem of having a meaning in life is that it can be lost. One way of expressing it is that we become lost in ourselves; we do not live in accordance with our deepest self. Deep down, life feels empty. Perhaps we do not want to get up in the morning. Or we may contemplate suicide. Meaninglessness seems to be a frequent reason why about 1,400 people commit suicide in Denmark per year.

The meaning of life is the theme of classical religion, and the world religions can be seen as theories on the meaning of life. For instance, the classical goal of Hinduism is to attain the experience of unity with the world, tat tvam asi – (you are it) and the highest meaning in life. In Buddhism, the highest goal is emptiness or nirvana, which centers on being at one with the deepest meaning in the world. Many Native American tribes see it as their ultimate goal to find their own wholeness. In Christianity, the message of the love of God leads to the central meaning in life.

The depths of our being, the distance between the surface and the deeper layers, leaves ample room for what we term life lies. They hide the depth of our existence. In our culture we collectively have one set of life lies that make meaning in life in its deepest sense a taboo. A theory for the meaning of life is the life mission theory[11].

## A Biological View of the Quality of Life: the Biological Information System and the State of Existential Balance

This aspect of the quality of life concerns the fundamental, biological constitution of humans. From a biological point of view, a human is a living organism, a colony of cells that, through intense exchange of information, realizes a deep biological potential to create a human being.

When the formula for becoming a person, which is contained deep within our nature, has been realized in the structure of the human organism, then life is characterized by order; biologically, psychologically, socially, and religiously understood in a broad sense as what we believe about life and reality.

Viewed from this angle, physical health reflects the state of the biological information system, as the cells of the body need precise information to function correctly and to keep the body healthy and well. According to this notion, the quality of life lies in the conformity between the actual life lived and the formula for being a person that lies deep within the organism.

As our consciousness and life experiences are also biologically conditioned, the experience that life has or does not have meaning can also be seen as conditioned by the state of the biological information system. If communication between the cells of the organism is not optimal, states of experience and of conscious life cannot be optimal either.

Some people with arthritis are doing well and some people who are not sick are doing poorly. The interesting question is whether one can have arthritis and still have a life that is totally meaningful. If there is a connection between the quality of life and illness, it is probably located deep within human beings in connection with their existential center (including their state of health and "soul") and not on the surface in connection with how good they feel. Meaning in life and biological order go hand in hand in such a life theory (see Fig.1)[11,12,13].

The person who lives in such a way that their meaning in life is gradually lost is also the kind of person who is susceptible to illnesses that affect the physical appearance and well being of the body, seemingly without cause. The relation between the quality of life and illness is therefore best illustrated by using a theory of the individual as a biological information system.

## Realizing Life Potential

Humans are in constant development. It starts with a fertilized egg, which contains a vast array of information. Throughout life, it must manifest and realize the potential of this information. Taking this as our starting point, realizing life potential becomes a key concept in the quality of life.

Just as a sunflower seed uses its potential to become a sunflower with flowers and leaves, humans use a store of potential for creative activities, good social relations, a meaningful job, and starting a family: living life to the full.

In the course of realizing our life potential, we all link with reality on a number of levels. The central nervous system — the brain — enabled us to arise from the earth in which we were planted and relocate to another place better suited for self-realization. Human life is thus far more complex than that of the sunflower. It develops in far more abstract ways.

The theory of realizing life potential is a theory of the links between nature and humanity. It links the quality of life closely to the human roots in nature. This biological starting point does not reduce the significance of living beings but merely states that this is a general theory of the exchange of meaningful information in living systems: from cell to organism to society (a biopsychosocial model). This theory is explained in detail in the next paper in this series[14].

## Fulfillment of Needs

The concept of fulfillment of needs is far less abstract than the previous aspects. It is also more superficial and culturally conditioned. It is used in a broad sense, also popularly. The needs traditionally relate to the quality of life such that, when your needs are fulfilled, your quality of life is high. The needs are an expression of our nature: that is, something all human beings have in common.

The notion of fulfillment of needs is ubiquitous: "I need a cup of coffee" or "We all need the security a family can give us." The concept is used far more stringently in scientific terms because a closer look reveals that the meaning of the concept is unclear and ambiguous.

Maslow[13] provided a very important formulation of the concept of needs. Besides such well-known needs as food, sex, and social relations, Maslow talks about a more abstract need to realize oneself. This idea has become an integral part of our culture.

As outlined above, the notion of fulfillment of needs is intuitively meaningful. It is a stage between the deep, extremely existential aspects and the superficial objective phenomena, and we have therefore included it in our spectrum. The need is also closely linked with desires and the satisfaction we all achieve by having our needs fulfilled. The subjective part of the spectrum includes satisfaction and is intermediate between the superficial aspects and the depths of our being.

Fulfillment of needs is not identical to well being, as needs are related to aspects of human nature. However, it is part of the theory of needs that we feel good once our needs have been fulfilled. Fulfillment of needs is not the same as realizing life potential, which takes the extreme stance that life is all about realizing one's biological information. This information exists in such complex forms that one can hardly reduce it to simple, actual needs.

If we take Maslow's need in the abstract, the human need for self-realization, the definition becomes vague and unclear. A need is no longer something the individual has to satisfy from an outside source such as food and shelter but something the person has to contribute to and accomplish for himself or herself. Are we then to fulfill this urge from the outside world or from within our inner depths? And does this then mean that our needs are fulfilled? Who or what in each of us, is it that needs? The ego? The self? Learned expectations?

The more one digs into the concept of need, the more unclear and ambiguous it all seems. Many researchers, notably sociologists, suggest that we should move away from talking of need[15]. Nevertheless, the need concept is extremely simple to work with in practice because the concept is so well integrated into many languages. The third paper in this series present a modern understanding of Maslow[16].

## Objective Factors

The objective aspects of the quality of life are related to the external factors of life and are fairly easy to establish. They include income, marital status, state of health, and the number of daily contacts with other people.

We need to distinguish these aspects of the quality of life, because a good life is easily confused with the sort of life we generally consider as being "right", and rich in an external sense of the word. You can be unhappy in a sports car and happy in a tram car. This general truth is lent credence by the clinical fact that there is often little agreement between a physician's evaluation of a person's (objective) quality of life and the patient's own evaluation of his or her (subjective) quality of life.

The factors chosen to indicate the objective quality of life are closely associated with the culture in which we live. In Denmark, most people believe that two children are sufficient to give us maximum quality of life. Another culture would seem to think that two or three is not enough. The objective quality of life thus very much reflects our ability to adapt to the culture in which we live. It is thus somewhat superficial, as it does not presuppose any deep reflection or pondering on the nature of this culture. The degree of adaptation to the norms of the culture is superficially synonymous with the notion of well being.

Research into the quality of life has been greatly influenced by attempts to measure the quality of life objectively. These tests span from the purely objective from a medical viewpoint (Can you stand? Walk? Run?) to counting, by sociologists, of people's social contacts, etc. (state-of-life research). Practically all such research is based on theories that generate lists of the objective factors that are part of a good life. This can be done in a number of ways. Ours is based on the cultural orientation of our Western societies and on the theory of realizing life potential.

## THE INTEGRATIVE THEORY AND THE DEPTHS OF LIFE

All these theories are aspects of life that can be placed on a horizon moving from the subjective to the objective. Number 1, well being, and number 8, objective factors, are the most superficial, as they are concerned with our superficial ability to adapt to our culture. Number 2, satisfaction with life, and number 7, fulfillment of needs, are concerned with somewhat deeper aspects: is there symmetry between what I want from life and what life gives me? Number 3, happiness, and number 6, realizing life potential, encompass our deepest existence and nature as individuals. Number 4, meaning in life, and number 5, order and disharmony in the biological information system, deal with humanity's innermost being.

We need to also imagine an abstract nucleus between 4 and 5 that remains inexpressible and immeasurable. The subjective and the objective unite in this nucleus: the deepest subjective quality of life and the objective state of the organism, as we know it from health and sickness. There, deep down in the individual, at the very center where meaning in life is created, lies our innermost pool, the very center of our existence (see Fig. 1).

How the individual feels here, at the very center of humanity, must reflect the quality of life, the deepest knowledge of humanity we can attain. Unfortunately, it is probably impossible to express this state in words, because the experience does not belong to the world of logic and order but to the irrational, life's own space and quality, through an experience that is present and spontaneous and beyond any dimension that would be required of rational description and analysis.

One might choose other words for this deepest pool, which is what great religious, mystical, and philosophical systems do. They all reckon with the occurrence of a depth in life from which all that is truly valuable in our existence originates. No matter what one calls this depth, life is the ability to contact it and bring it in accordance with what each of us considers to be the true essence of life. If we believe sufficiently in ourselves, we can bring forth this essence from the depths of our being and let it be part of our own lives.

## CONCLUSIONS

The philosophy of life outlined in this paper is that to measure the global quality of life, we must do this according to an integrative theory of the quality of life, such as the IQOL theory. The IQOL theory is an overall theory or meta-theory encompassing eight more factual theories in a subjective-existential-objective spectrum. Other philosophies of life and other concepts of QOL can stress other aspects of life (and do so)[17]. We must not presuppose any consensus on the nature or on the existential depths of our being, which each and every one of us possesses. But the very notion of introducing such a depth into the health and social sciences would seem to be a necessary step towards a new humility and respect for the richness and complexity of life.

## ACKNOWLEDGMENTS

This study was supported by grants from The 1991 Pharmacy Foundation, as well as by supplementary grants from Goodwill-fonden, the JL-Foundation, E. Danielsen and Wife's Foundation, Emmerick Meyer's Trust, the Frimodt-Heineken Foundation, the Hede Nielsen Family Foundation, Petrus Andersens Fond, Wholesaler C.P. Frederiksens Study Trust, Else & Mogens Wedell-Wedellsborg's Foundation, and IMK Almene Fond. We gratefully acknowledge the critical scrutiny and expert linguistic assistance of Ib Ravn, Ph.D. The research was approved by the Copenhagen Scientific Ethical Committee under number (KF)V.100.2123/91.

## BIOSKETCHES

**Søren Ventegodt, MD,** is the Director of the Quality of Life Research Center in Copenhagen, Denmark. He is also responsible for a Research Clinic for Holistic Medicine in Copenhagen and is a popular speaker throughout Scandinavia. He has published numerous scientific or popular articles and a number of books on holistic medicine, quality of life, and quality of working life. His most important scientific contributions are the comprehensive SEQOL questionnaire, the very short QoL5 questionnaire, the integrated QOL theory, the holistic process theory, the life mission theory, and the Danish Quality of Life Research Survey, 1991–94 in cooperation with the University Hospital of Copenhagen and the late pediatric professor Bengt Zachau-Christiansen. E-mail: ventegodt@livskvalitet.org. Website: www.livskvalitet.org/

**Joav Merrick, MD, DMSc,** is Professor of Child Health and Human Development affiliated with the Zusman Child Development Center and Division of Community Health at the Ben Gurion University, Beer-Sheva, Israel and presently the Medical Director of the Division for Mental Retardation, Ministry of Social Affairs, Jerusalem and the Director of the National Institute of Child Health and Human Development. He has numerous publications in the field of child and human development, rehabilitation, intellectual disability, disability, health, welfare, abuse, advocacy and prevention. Dr. Merrick received the Peter Sabroe Child Award for outstanding work on behalf of Danish Children in 1985 and the International LEGO-Prize ("The Children's Nobel Prize") for an extraordinary contribution towards improvement in child welfare and well being in 1987. E-mail: jmerrick@internet-zahav.net. Website: www.nichd-israel.com

**Niels Jørgen Andersen, MSc,** Professor, Department of Innovation and Economic Organization, Norwegian School of Management. This department conducts research and provides teaching in central topics related to innovation, business development, management of global companies, business history, and economic organization. Research activities within the Department are related to four core subjects within the discipline: business history, cooperative organizations, business development and entrepreneurship, and finally studies of industries with a special focus on the electricity industry. He is also the dynamic chairman of the nonprofit organization Stiftelsen Holistisk Medisin Scandinavia, which aims to support the scientific development, research, and documentation of complementary and holistic medicine in Scandinavia. E-mail: niels.j.andersen@bi.no. Website: www.bi.no/users/fgl93013/
